# Effectiveness of Tocilizumab in juvenile patients with refractory Takayasu arteritis: Two case reports

**DOI:** 10.1097/MD.0000000000018890

**Published:** 2020-01-24

**Authors:** Tomoyuki Asano, Shuzo Sato, Jumpei Temmoku, Yuya Fujita, Makiko Yashiro Furuya, Naoki Matsuoka, Hiroko Kobayashi, Eiji Suzuki, Hiroshi Watanabe, Kiyoshi Migita

**Affiliations:** a Department of Rheumatology, Fukushima Medical University School of Medicine, Hikarigaoka, Fukushima; b Department of Rheumatology, Ohta Nishinouchi General Hospital, Koriyama, Fukushima, Japan.

**Keywords:** interleukin-6, intermittent claudication, serum amyloid A, Takayasu arteritis, tocilizumab

## Abstract

**Rationale:**

Takayasu arteritis (TA) is a systemic large-vessel vasculitis which can be accompanied by the symptoms associated with vascular stenosis.

**Patient concerns:**

We describe 2 female juveniles with TA who presented with progressive intermittent claudication.

**Diagnosis:**

Contrast-enhanced computed tomography (CT) revealed the stenosis of femoral arteries and increased levels of C-reactive protein (CRP), and serum amyloid A (SAA) were noted in both patients. According to European league against rheumatism consensus criteria for the diagnosis of TA was confirmed in both patients.

**Interventions:**

Both patients had shown resistance to glucocorticoids and treated with tocilizumab (TCZ) (subcutaneous injections, 162 mg/week).

**Outcomes:**

These treatments improved claudication symptoms. Follow-up imaging by enhanced CT revealed restoration of advanced stenosis of the femoral arteries in both patients. They achieved normalization of levels of the acute-phase reactants CRP and SAA. Serum levels of interleukin-6 were increased transiently after TCZ injection, but declined to within normal ranges at 12 weeks.

**Lessons:**

Juvenile patients with TA presenting with advanced stenosis of the femoral arteries are not rare. The clinical courses of our patients suggested the beneficial effects of TCZ against the progressive vascular stenosis observed in refractory TA.

## 1 Introduction

Takayasu arteritis (TA) is a chronic inflammatory disorder of unknown etiology that involves large and medium-sized arteries.^[1]^ Without precise control of TA, chronic, and progressive inflammation of vessels leads to vascular stenosis, followed by end-organ ischemia, which is associated with significant morbidity and mortality.^[2]^ After the diagnosis of TA, standard immunosuppressive therapy including high-dose glucocorticoids (GCs), should be started with or without another immunosuppressive agent.^[3]^ In TA patients with clinical manifestations of vascular stenosis, revascularization procedures are also required.^[4]^ Immunosuppressants or biologic agents have shown beneficial effects against progressive vascular inflammatory lesions in combined with GCs therapy in small observational studies.^[5]^ However, studies focusing on the therapeutic efficacy of immunosuppressants or biologic agents against advanced vascular stenosis are lacking. We describe 2 juvenile causes of TA presenting with progressive stenosis of large vessels, which were successfully managed by tocilizumab (TCZ) treatment.

## 2 Case report

### 2.1 Case 1

An 18-year-old woman visited our hospital with a 1-year history of TA with intermittent claudication of the left leg. She presented with low-grade fever in 1 month prior to the current visit. There was no history of dyspnea, palpitations, dizziness, visual disturbances or carotidynia. She visited a local hospital and also reported claudication of the left leg. Clinical examination revealed bruits over the left femoral arteries, and laboratory data indicated increased levels of C-reactive protein (CRP). She was diagnosed with TA according to the European League Against Rheumatism (EULAR) criteria for TA.^[6]^ Initially, she was treated with oral prednisolone (PSL) (40 mg/day) and the increased levels of CRP were normalized. Finally, PSL was tapered to 10 mg/day. She was referred to our hospital for maintenance therapy due to movement problems. In the first visit to our hospital, intermittent claudication improved partially, however she recognized claudication symptoms after walking more than 100 m.

**Figure 1:**
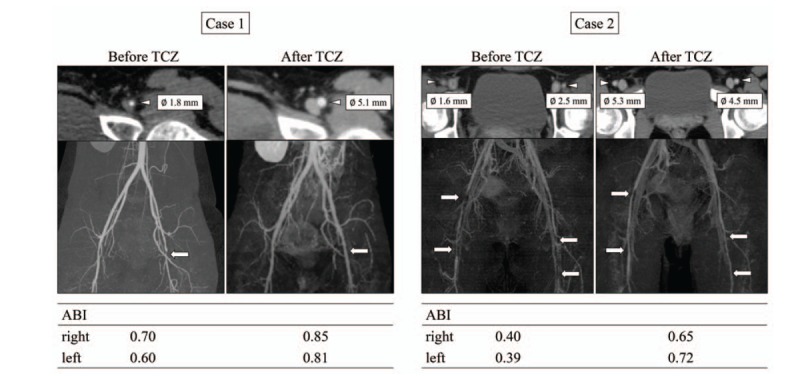
Clinical course and circulating IL-6 levels during treatment with tocilizumab (case 1). Serum levels of interleukin (IL)-6 measured by a Human IL-6 ELISA kit (R&D Systems, Minneapolis, MN). CRP = C-reactive protein, ELISA= enzyme linked immunosorbent assay, IL-6 = interleukin-6, SAA = serum amyloid A, TCZ = tocilizumab.

Upon physical examination, the pulse rate was 88 bpm. Radial pulses were normal and equally palpable on both sides. A bruit was audible over the inguinal region on the left side without any carotid, renal or abdominal bruits. Hematology and biochemistry (Table 1) revealed no abnormality except increased serum amyloid A (SAA) (53.4 μg/mL) and CRP level (1.26 mg/dL). Antinuclear antibodies (ANAs) were positive with low titers (1:80), and anti-neutrophil cytoplasmic antibodies (ANCAs) were negative. Enhanced computed tomography (CT) showed the stenosis of the left femoral artery (Fig. 1, case 1).

**Table 1:**
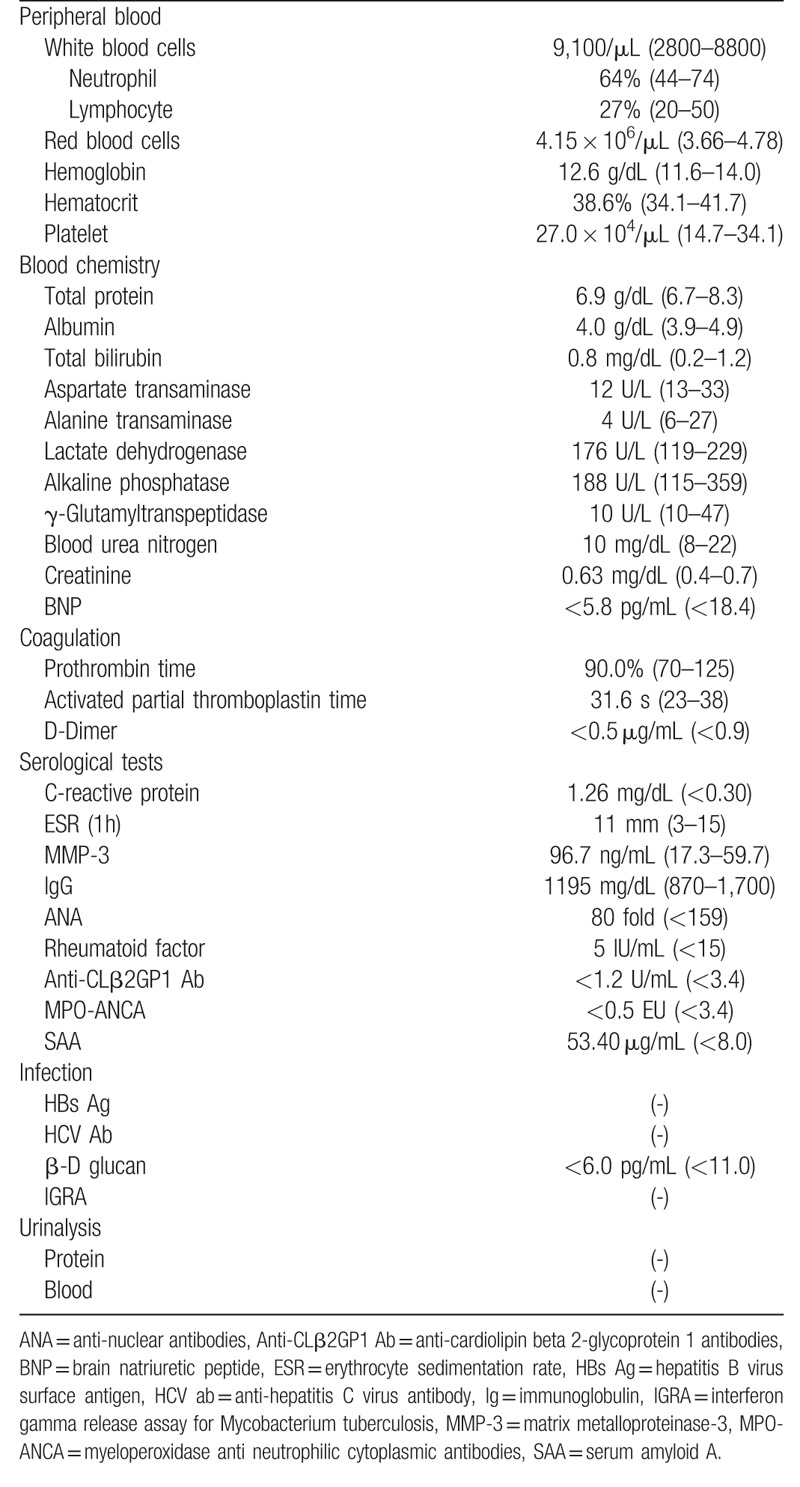
Laboratory findings on admission.

**Figure 2:**
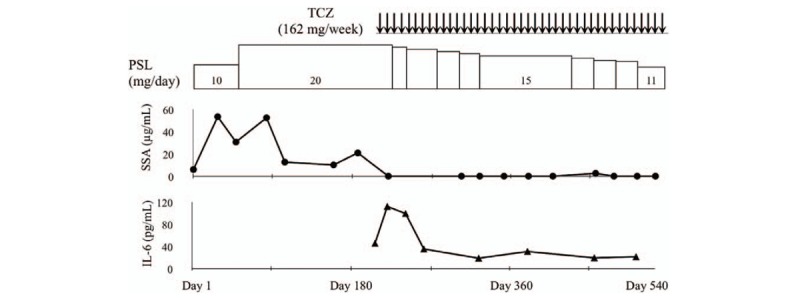
Computed tomography findings on femoral arteries, and ankle-brachial index before and after tocilizumab therapy. Three-dimensional (3D) construction images of enhanced computed tomography showing marked stenosis of femoral arteries. Follow-up CT at 6 mo from the start of tocilizumab treatment showed resolution of the obstructive lesions in femoral arteries. Arrow indicates high-grade stenosis of femoral arteries. The femoral artery diameter was measured. The stenosis was dilated after TCZ treatment. ABI = ankle–brachial index, TCZ= tocilizumab.

Owing to sustained intermittent claudication and relapse of increased levels of CRP, additional immunosuppressive treatments were required. We decided to increase the dose of PSL (10 mg/day → 20 mg/day), and the increased levels of CRP fell to within normal ranges. However, intermittent claudication was not controlled completely by GCs therapy, and elevated levels of SAA (20.7 μg/mL) were still observed. Hence, we introduced TCZ treatment in combination with maintenance GCs therapy. We tried to taper the PSL dose under the concomitant TCZ treatment with stable dose (subcutaneous injections, 162 mg/week). Serum levels of SAA were normalized (<2.5 μg/mL) within 3 weeks from the start of TCZ treatment. During this treatment periods, PSL dose was gradually tapered. Six months after starting TCZ, contrast-enhanced CT revealed a marked reduction of stenosis in the left femoral artery. Also, the ankle–brachial index (ABI) showed an increase in blood pressure of the lower extremities (Fig. 1, case 1). One year after starting TCZ treatment, the patient remained asymptomatic even after walking >2 km and there are no relapsing signs of TA. Serum levels of interleukin (IL)-6 (measured using a Human IL-6 ELISA kit; R&D Systems, Minneapolis, MN) showed high levels (27.5 pg/mL) before initiation of TCZ treatment. Serum levels of IL-6 increased transiently, but declined to the lower levels (10–20 pg/mL) at 12 weeks from the start of TCZ treatment (Fig. 2). TCZ treatment had been continued and there was no TCZ-related adverse effect at the time of this writing. Doses of PSL were tapered gradually to the maintenance dose (11 mg/day), which was accompanied without disease flare.

### 2.2 Case 2

A 16-year-old woman presenting with low-grade fever, general fatigue, headache and intermittent claudication was referred to our hospital. One year before the current episode, she had suffered from headache, increasing weakness, and claudication in both legs. She had visited a local hospital, and the increased levels of erythrocyte sedimentation rate and CRP were pointed out. Ultrasonography of the cervical region was undertaken to look for inflammatory changes. It revealed a characteristic, homogeneous and circumferential thickening of the wall of the entire left common carotid artery. She was referred to our hospital for further examination.

Upon admission, physical examination revealed discrepancies in blood pressure (in mmHg) in the right arm (129/53), left arm (136/58), right leg (54/27) and left leg (53/37). Laboratory data (Table 2) revealed anemia (Hemoglobin 10.5 g/dL) and increased CRP concentration (4.1 mg/dL). ANAs were positive with low titer (1:160), but ANCAs were not detected. Enhanced CT revealed significant thickening of the aortic-artery wall and both carotid arteries. Also, there was severe stenosis of both femoral arteries (left >right) originating from intermittent claudication (Fig. 1, case 2).

**Table 2:**
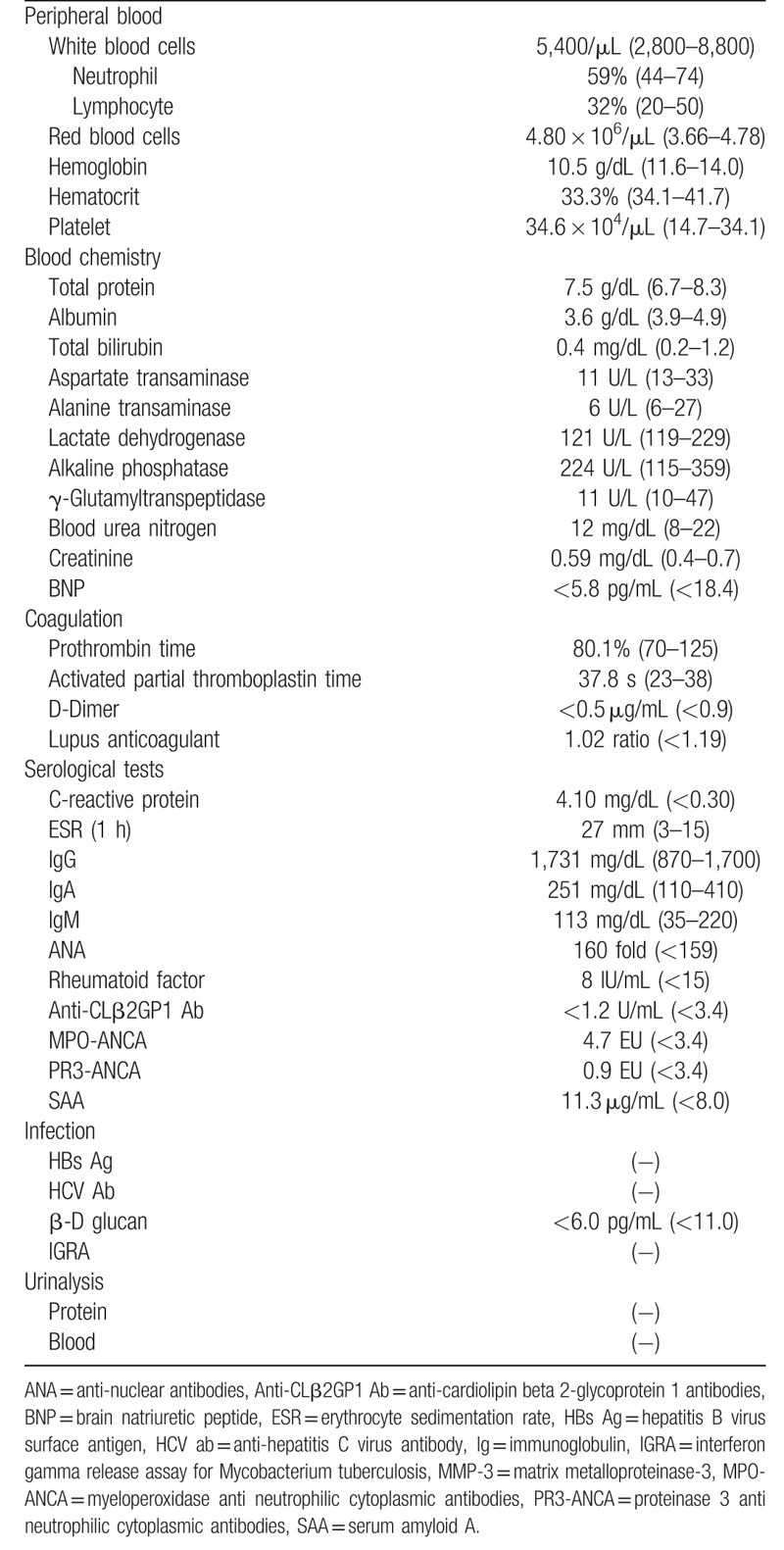
Laboratory findings on admission.

The patient was diagnosed as TA according to the EULAR criteria for TA.^[6]^ She was started on PSL (50 mg/day) and antiplatelet therapy (cilostazol, 200 mg/day). The CRP concentration and ESR returned to normal values, and her intermittent claudication was improved partially. However, serum levels of SAA were not normalized (11.3 μg/mL). Therefore, we started TCZ (subcutaneous injections, 162 mg/week) therapy in combination with GCs treatment. After TCZ therapy, the increased levels of SAA declined to <2.5 μg/mL. Repeated imaging revealed significant improvement in the luminal diameter of the both femoral arteries, and arterial stenosis was decreased significantly. Also, the ABI showed an increase in blood pressure of the both lower extremities (Fig. 2 case 2). TCZ treatment had been continued with same dose, which permitted tapering of PSL to the maintenance dose (13 mg/day) over 17 months. There was no TCZ-related adverse effect at the time of this writing. Similar to case 1, elevated circulating IL-6 was declined (21.8 pg/mL → 3.2 pg/mL) at 12 weeks after the start of TCZ.

## 3 Discussion

TA is a rare, chronic vasculitis involving the aorta and its major branches that appears commonly at young age.^[1]^ To prevent the progression of large-vessel stenosis or occlusion, medical intervention plays important parts in the management of TA.^[7]^ In this case series, we reported the clinical responses to TCZ in 2 patients with TA presenting with rapidly progressive intermittent claudication. Repeated enhanced CT and ABI testing revealed a significant retraction of advanced stenosis of the femoral arteries.

The most common localization of vasculitis in TA in the aortic root and ascending aorta. However, TA affects all whole large vessels and their branches.^[1]^ Symptoms may range from systemic (eg, fever) to those caused by narrowing of the vascular lumen of the affected arteries.^[8]^ Therefore, intermittent claudication is one of the important symptoms predicting irreversible vascular occlusion of large blood vessels.^[9]^ GCs remains first-line treatment for TA, but a high prevalence of relapse during tapering of the GCs dose leads to administration of other immunosuppressive agents.^[1,3]^ In particular, juvenile-onset TA remains a therapeutic challenge because GCs and conventional immunosuppressants are not always efficacious.^[10]^

The number of immune cells and levels of cytokines (Tumor necrosis factor-α, IL-6, IL-8 and IL-17A) are increased in patients with TA.^[11]^ Hoffman et al reported the use of tumor necrosis factor-α (TNF) inhibitors against refractory TA, and 10 of their 15 patients achieved complete remission without GCs use.^[12]^ The colleagues reported on the long-term efficacy of 25 patients with refractory TA treated with TNF inhibitors.^[13]^ Remission was achieved without GCs use in 15 patients, and GCs were tapered to <10 mg/day in 7 patients (28%).^[13]^ Circulating IL-6 levels have been shown to be associated with TA activity.^[14]^ Therefore, IL-6 could be a therapeutic target in refractory TA. TCZ is a humanized anti-IL-6 receptor monoclonal antibody reported to be effective against refractory TA.^[15]^ It was also reported that thickening of the aortic wall diminishes after TCZ therapy in patients with refractory TA.^[16]^ High dose of GCs usually initiated as an induction therapy for active TA. Whereas we used TCZ as a GCs-sparing agent on the basis of published data^[17]^ or EULAR recommendation for the management of large vessel vasculitis.^[18]^ In cases of refractory TA with symptoms of ischemia or progressive vascular inflammation, high doses of GCs or initiation of adjunctive therapy are recommended.^[18]^ Recent randomized clinical trial confirmed the effectiveness of TCZ as GCs-sparing intervention in Japanese patients with TA.^[17]^ These emerging reports suggest a pivotal role of IL-6 in TA pathogenesis, and further investigations are merited. However, there is little evidence for the use of biologics against refractory TA with progressive vascular stenosis of large vessels. Our 2 cases suggest that IL-6 blockade may be a beneficial therapeutic option in TA patients with advanced stenosis of large vessels. Serum levels of SAA were demonstrated to be normalized after 1 month of TCZ treatment which was associated with the clinical improvements, such as reduction in the thickening of the wall of large-vessels.^[16]^ In consistent with this previous report, serum levels of SAA were normalized by TCZ treatment. Although we present evidence for only 2 cases, we propose that IL-6 blockade is an alternative treatment in refractory TA with progressive vascular damage.

The serum IL-6 level could serve as a predictive marker for remission in rheumatoid arthritis patients receiving TCZ.^[19]^ This strategy is valid because the serum level of IL-6 under TCZ treatment would reflect the actual level of endogenous IL-6 production that correlates with the level of “true” disease activity using the “bathtub model” postulated by Nishimoto et al^[20]^ Consistent with the bathtub model, after the induction IL-6 blockade using TCZ, a temporary increase in serum IL-6 levels was noted and, subsequently, IL-6 levels were normalized during continued TCZ treatment (at 12 weeks) in our cases. These findings suggest that the serum IL-6 level might be a useful biomarker for evaluating progressive vascular damage and the therapeutic efficacy of TCZ in TA patients.

## 4 Conclusions

This case series demonstrated the restorative effects of TCZ against progressive vascular stenosis in TA. It also highlighted a crucial role of IL-6 in perpetuating the progressive vascular stenosis observed in TA. Further exploration of the role of IL-6 for estimating ongoing large-vessel stenosis in refractory TA is required.

## Author contributions

**Conceptualization:** Tomoyuki Asano, Shuzo Sato, Makiko Yashiro Furuya, Jumpei Temmoku, Yuya Fujita, Naoki Matsuoka, Hiroko Kobayashi and Hiroshi Watanabe.

**Supervision:** Kiyoshi Migita.

**Writing – original draft:** Tomoyuki Asano and Kiyoshi Migita.

**Writing – review & editing:** Kiyoshi Migita

## References

[1] Mason JC Takayasu arteritis--advances in diagnosis and management. Nat Rev Rheumatol 2010;6:406–15. 20596053 10.1038/nrrheum.2010.82

[2] Maksimowicz-McKinnon K Clark TM Hoffman GS Limitations of therapy and a guarded prognosis in an American cohort of Takayasu arteritis patients. Arthritis Rheum 2007;56:1000–9. 17328078 10.1002/art.22404

[3] Clifford A Hoffman GS Recent advances in the medical management of Takayasu arteritis: an update on use of biologic therapies. Curr Opin Rheumatol 2014;26:7–15. 24225487 10.1097/BOR.0000000000000004

[4] Pariser KM Takayasu's arteritis. Curr Opin Cardiol 1994;9:575–80. 7987037

[5] Schäfer VS Zwerina J Biologic treatment of large-vessel vasculitides. Curr Opin Rheumatol 2012;24:31–7. 22089099 10.1097/BOR.0b013e32834dc392

[6] Ozen S Pistorio A Iusan SM EULAR/PRINTO/PRES criteria for Henoch-Schönlein purpura, childhood polyarteritis nodosa, childhood Wegener granulomatosis and childhood Takayasu arteritis: Ankara 2008. Part II: final classification criteria. Ann Rheum Dis 2010;69:798–806. 20413568 10.1136/ard.2009.116657

[7] Wen D Du X Ma CS Takayasu arteritis: diagnosis, treatment and prognosis. Int Rev Immunol 2012;31:462–73. 23215768 10.3109/08830185.2012.740105

[8] Alibaz-Oner F Aydin SZ Direskeneli H Advances in the diagnosis, assessment and outcome of Takayasu's arteritis. Clin Rheumatol 2013;32:541–6. 23271611 10.1007/s10067-012-2149-3

[9] Dong H Che W Jiang X An unrecognized presentation of Takayasu arteritis: superficial femoral artery involvement. Clin Exp Rheumatol 2017;103:83–7. Suppl. 27908309

[10] Aeschlimann FA Eng SWM Sheikh S Childhood Takayasu arteritis: disease course and response to therapy. Arthritis Res Ther 2017;19:255 10.1186/s13075-017-1452-4. 29166923 10.1186/s13075-017-1452-4 PMC5700506

[11] Terao C Yoshifuji H Mimori T Recent advances in Takayasu arteritis. Int J Rheum Dis 2014;17:238–47. 24548718 10.1111/1756-185X.12309

[12] Hoffman GS Merkel PA Brasington RD Anti-tumor necrosis factor therapy in patients with difficult to treat Takayasu arteritis. Arthritis Rheum 2004;50:2296–304. 15248230 10.1002/art.20300

[13] Molloy ES Langford CA Clark TM Anti-tumour necrosis factor therapy in patients with refractory Takayasu arteritis: long-term follow-up. Ann Rheum Dis 2008;67:1567–9. 18677012 10.1136/ard.2008.093260

[14] Yoshifuji H Pathophysiology of large vessel vasculitis and utility of interleukin-6 inhibition therapy. Mod Rheumatol 2019;29:287–93. 30427262 10.1080/14397595.2018.1546358

[15] Nishimoto N Nakahara H Yoshio-Hoshino N Successful treatment of a patient with Takayasu arteritis using a humanized anti-interleukin-6 receptor antibody. Arthritis Rheum 2008;58:1197–200. 18383395 10.1002/art.23373

[16] Nakaoka Y Higuchi K Arita Y Tocilizumab for the treatment of patients with refractory Takayasu arteritis. Int Heart J 2013;54:405–11. 24309452 10.1536/ihj.54.405

[17] Nakaoka Y Isobe M Takei S Efficacy and safety of tocilizumab in patients with refractory Takayasu arteritis: results from a randomised, double-blind, placebo-controlled, phase 3 trial in Japan (the TAKT study). Ann Rheum Dis 2018;77:348–54. 29191819 10.1136/annrheumdis-2017-211878 PMC5867398

[18] Hellmich B Agueda A Monti S 2018 Update of the EULAR recommendations for the management of large vessel vasculitis. Ann Rheum Dis 2019;79:19–30. 31270110 10.1136/annrheumdis-2019-215672

[19] Shimamoto K Ito T Ozaki Y Serum interleukin 6 before and after therapy with tocilizumab is a principal biomarker in patients with rheumatoid arthritis. J Rheumatol 2013;40:1074–81. 23637318 10.3899/jrheum.121389

[20] Nishimoto N Terao K Mima T Mechanisms and pathologic significances in increase in serum interleukin-6 (IL-6) and soluble IL-6 receptor after administration of an anti-IL-6 receptor antibody, tocilizumab, in patients with rheumatoid arthritis and Castleman disease. Blood 2008;112:3959–64. 18784373 10.1182/blood-2008-05-155846

